# Managing snacking behaviors through energy density, portion control, sensory properties, and packaging

**DOI:** 10.3389/fnut.2026.1919469

**Published:** 2026-09-17

**Authors:** Federico Canzoneri, Adam Drewnowski, Barbara J. Rolls, Francesco Tramontin, Emanuela Cavalli

**Affiliations:** 1 Ferrero Group, Soremartec Italia Srl, Alba, Italy; 2 Center for Public Health Nutrition, University of Washington, Seattle, WA, United States; 3 Department of Nutritional Sciences, The Pennsylvania State University, University Park, PA, United States; 4 Ferrero Group, Ferrero International SA, Senningerberg, Luxembourg

**Keywords:** energy density, packaging, portion size, snacking, structured consumption moments, taste and texture

## Abstract

Between-meal snacks contribute >20% of daily energy intakes to typical Western diets. Most snacks are both energy-dense and palatable. This perspective summarizes the scientific foundation of responsible snacking, focusing on energy density, portion control, innovative packaging, taste and texture, and intentional consumption. Laboratory studies show that pairing energy-dense snacks with fruits or whole grains helps to reduce energy density of the eating occasion, leading to fewer calories consumed. Reducing serving sizes of energy-dense foods is another laboratory tested strategy for calorie management. Individually packed and wrapped snacks have helped manufacturers restrict snack calories to 150–200 kcal per serving. Innovations in snack taste and texture can slow eating rate and promote greater enjoyment at fewer calories. Finally, attentive eating can trigger commensal social mechanisms for portion control. The Dietary Guidelines for Americans (DGA) and professional associations have always noted that healthy diets can include both meals and between-meal snacks. The current DGA 2025–30 specifically recommend that both children and adults consume smaller portions of energy-dense foods to stay within daily energy requirements, very much in line with the present perspective. However, the responsibility for assuring healthier snacking also falls on food manufacturers. Consumer-facing behavioral strategies for responsible consumption need to be complemented by manufacturer-led initiatives in such areas as individually packaged portions, product formulation, transparency, and clear labeling.

## 1 Introduction

Between-meal snacks have become an integral component of modern eating habits (1–5). Snacks are defined as eating events occurring outside the traditional meals: breakfast, lunch or dinner. Based on current estimates, snacks now provide about 20–22% of daily dietary energy in the United States, that is more calories than either breakfast, or lunch, or dinner (4). Energy contribution of snacks has been reported to be lower in France (~14%) (6) and Australia (~13%) (7) than in the US, with intermediate values reported in Mexico (~17%) (8) and Norway (men 17%; women 21%) (9). Even higher estimates have been observed in Finland (10). While both solid foods and beverages can be consumed as snacks, the focus of the present perspective is on solid snacks. In the most recent National Health and Nutrition Examination Survey (NHANES) in the US, solid snacks, mostly energy dense, accounted for about 16% of daily energy (4).

Given the importance of snacks in everyday diets, we need innovative behavioral strategies to manage responsible consumption. This Perspective lists some science-backed strategies for integrating snacks into more healthful diets. The present goal is to suggest strategies that are directly related to the current food environment rather than promote goals that may not be feasible in most environments. Whereas some strategies are consumer-facing, others suggest potential actions by food manufacturers.

The notion that healthy eating patterns can include both meals and snacks is well established in the Dietary Guidelines for Americans (DGA) (11). The DGA 2020–25 recommended that consumers select healthy foods, beverages, meals, and snacks specific to their needs and preferences (11). One prevailing view has been that snacks should be low energy density foods that would not reduce appetite for a “proper” meal (11). Referring to the need for more energy-dense snacks, the Academy of Nutrition and Dietetics (12) noted that snacks can fit into a healthy eating plan and provide an energy boost between meals, if they're planned right.

There are many reasons why people snack. Consumers report snacking to alleviate hunger, provide an energy boost between meals, manage low blood sugar levels, and improve physical and mental performance (13). Beyond hunger and physiological needs, snacking behavior can be influenced by a range of emotional and contextual factors, such as boredom, job monotony, psychological stress, and work-related time pressure (14). Marketing studies report that consumers seek out those snacks that are good-tasting, affordable, accessible, and convenient (15). Common energy dense snacks include nuts, seeds, dried fruit, whole grain bars, and chocolate (4). Manufacturers operating in this category need to find ways to support responsible snacking while continuing to meet consumers' expectations for taste, enjoyment, and convenience. We need innovative behavioral strategies to manage excess calories and help integrate energy-dense snacks into healthful diets.

It is important to note at the outset that the responsibility for managing responsible snacking lies both with the manufacturer and the consumer. All too often, snacking behavior is framed as a matter of individual choice. Yet consumer behavior is shaped by physiology, economics, and the environment. On one hand, eating behavior is driven by innate taste responses, food preferences, appetite, satiation and satiety. On the other hand, food manufacturers control snack formulation, portion size, packaging, pricing strategies, marketing practices, and the communication of product value to the consumer. Our perspective is that responsible snacking is not solely an individual concern but also a question of corporate responsibility. Here, we propose some science-backed strategies to help companies design new products and shape social contexts that support more balanced and healthier food patterns.

Effective dietary guidance would benefit from science-backed strategies for implementation. The three main behavioral strategies that we propose are backed by well-established studies in experimental psychology, sensory science, and sociology. As summarized in Table 1, the three consumer-facing strategies can be described as managing energy density and portion size, attending to sensory properties, and intentionally engaging in structured consumption moments. The sections that follow explore each principle in detail and outline some actionable strategies that manufacturers can adopt to foster responsible snacking behaviors. Proposed strategies for promoting responsible snacking for potential adoption by manufacturers are outlined in Table 2.

**Table 1:** The three main consumer-facing strategies for managing snacking behaviors.

**• Managing energy density and portion size:** Energy density and portion size are among the main drivers of energy intake. Pairing an energy dense snack with a lower energy density component (fruit, yogurt, whole grains) has been shown to reduce energy density of the eating occasion, leading to fewer calories consumed. Another approach is portion control, consuming smaller portions of energy-dense foods to stay within energy requirements (11). **• Attending to sensory properties of food:** Taste remains central to food choices. Snacks are formulated to deliver sensory experience and taste enjoyment while satisfying energy and nutritional needs. Attention to the positive sensory qualities related to snack taste and texture can slow down eating rate and promote greater satiation. **• Engaging in intentionally structured snacking moments:** Structured snacking, alone or in company, emphasizes attentive eating and a greater appreciation of sensory qualities of snacks. Commensality, the act of eating with others, can also result in soft regulation, expressed as social pressures to avoid overeating and limit portion size.

**Table 2:** Three actionable strategies for manufacturers to promote responsible snacking.

**• Managing energy density and portion size:** Reformulation initiatives have paired e.g., chocolate with dried fruit and whole grains, reducing the energy density of the product. Energy density has also been reduced by reducing the amount of sugar (in yogurt), by blending sugar with low calorie sweeteners (LCS) or by using LCS instead of sugar and by aeration. Fat content can be reduced by removing fat or using textures that mimic fat. Portion size of the snacks can be reduced through smaller, individually packaged servings. Manufacturers can support portion control by packaging, portion indications, visual cues, and labeling. **• Optimizing sensory properties and nutrient density:** Taste remains central to food choices. Positive sensory qualities related to snack taste and texture are achieved through purposeful product formulation. Promoting chewiness and viscosity can slow down eating rates. Snacks need to deliver taste enjoyment along with energy and nutrients. **• Transparency:** Manufacturers will need to develop nutrient profiles to serve as benchmarks and guides for product formulation and improvement.

## 2 Energy density and portion size management

### 2.1 Experimental studies on portion size

The importance of portion control in managing consumption varies depending on the energy density of the food consumed. Energy density is the ratio of calories to food weight (kcal/gram), with carbohydrates and protein contributing 4 kcal/g, and fat 9 kcal/g (16, 17). Water adds weight and volume to food but provides no calories. Foods with lower energy density such as fresh produce contain more food moisture (16, 17). By contrast, energy-dense foods are those foods that are dry and/or high in fat content.

Food weight, and to some extent food volume, affects satiety and plays a role in the regulation of food intake (18, 19). For low energy-density foods, reducing portion sizes has little impact on energy intake (19). Examples of low energy-density snacks that provide less than 1 kcal/gram are raw vegetables, whole fruit, and unsweetened yogurt. Larger portions of low energy-density foods promote volume-related fullness and provide satiation with relatively few calories. That is the basis of the Volumetrics effect (20), a well-established approach to calorie control and weight management. A larger weight (or volume) of low-energy-density foods eaten during a meal allows consumers to feel full on fewer calories. To better manage food consumption at a given meal, Rolls and Barnett (20) recommends beginning the meal with a low-energy-density soup or a salad.

When it comes to high energy-density foods, portion control is key. When presented with larger portions, people generally tend to eat more, regardless of whether they are hungry or not. Failing to promote volume-related fullness, larger portions of high energy-density foods generally lead to higher energy intakes (13, 21). Offered energy-dense foods instead of soup, participants in studies by Rolls et al. (22) showed less increase in fullness and consumed significantly more calories. Offering larger portions of energy dense foods, subjects consumed more calories, without a proportional increase in satiety (21). Portion sizes and energy density have an additive effect (23). Large portion sizes of energy-dense foods may, over time, result in energy overconsumption and weight gain.

Energy density and portion size are among the main drivers of energy intake (22–25). If managing calories is a goal, there are several approaches to responsible consumption of energy-dense snacks. One approach is to reduce the energy density of the eating occasion (if not of the snack itself) by pairing an energy-dense snack with a lower energy-dense component. Nutrient density of the snack itself can be improved by incorporating ingredients that increase fibers, proteins and micronutrients, for example, chocolate with grains or dried fruit, while keeping energy content per serving under control. Even small reductions in energy density of snacks or eating occasions can lead to reductions in energy intake, and, over time, help manage body weight (26).

The second approach is to reduce portion size of energy-dense snacks (25). When reducing energy density is not a viable option, portion control becomes the default strategy. Whereas limiting portions of low energy-dense foods has little impact on intakes, limiting portions of energy-dense-foods can be very effective (13, 19). The importance of advising consumers to reduce portions of energy dense foods was most recently featured in the latest Dietary Guidelines for Americans 2025–30 (27). The agencies adopted the recommendation of the 2025 Dietary Guidelines Advisory Committee (DGAC) that both children and adults consume smaller portions of energy dense foods to better manage daily energy intakes (28).

### 2.2 Manufacturer-led initiatives on serving size

These lab-generated concepts have been confirmed by studies of manufacturer-led initiatives. Georgetown University's Portion Balance Coalition (29) asserts that offering confectionery in smaller, portion-controlled sizes is an effective method for managing calories. The Power of Portions report (29) states that reducing portions can imperceptibly nudge people to eat less. Research indicates that some consumers prefer and actively seek smaller portion sizes and many already purchase single-serve options. The findings from laboratory studies on portion control can be adapted to manufacturing practices. Packaged solid snacks can be individually portioned for each eating occasion. Offering snacks in small, individually packaged servings is an effective strategy for portion control (30). Individual packaging of snacks introduces mechanical barriers that promote greater awareness of the amounts consumed. Here snack packaging introduces a natural pause that provides an opportunity for self-awareness and restraint (31).

Behavioral studies show that individual packaging of single-serve snacks can precisely define the amount to be consumed, with less reliance on hunger cues. A recent systematic review found that food packaging influenced food consumption in short-term intake studies, with 36 of 40 studies listed reporting changes in energy intake (32). Such factors as smaller packaging size, partitioning, resealability, and on-pack imagery helped to reduce consumption of high-energy-density foods, supporting the principle of portion control. Features of food packaging can also help promote more nutrient rich food choices (33).

Studies conducted on chocolate snacks showed that unwrapped chocolates tend to be eaten in higher quantities than those that are wrapped and individually packaged (34). Opening each individual package, and the attendant pause, were reported to provide cues as the number of items being consumed. Additional consumer behavior studies suggest that opening individually wrapped pieces portion requires a brief moment of effort to unwrap each unit, which can prompt reflection and slow down eating rate (35). Studies show that smaller, individually packaged items lead to reduced consumption because each decision to unwrap and eat another piece demands attention, interrupting the flow of automatic eating (34). Empty wrappers also serve as visible cues, signaling how much has already been consumed (36).

Other features of packaging can provide clear visual cues indicating the suggested portion (34). Practical solutions include using tray dividers, printed portion indicators on packaging, or contrasting colors to visually separate recommended amounts. Front of pack labels indicating the number of portions and calories per serving size can be very helpful in this respect.

In summary, manufacturer-determined portion size can act as a powerful behavioral nudge. Acting in parallel with clear labeling, package sizes can impact consumption through automatic, unconscious decision-making processes. Individually packaged smaller snacks are a step toward managing responsible snacking.

## 3 Sensory properties of snacks

Taste is the main driver of food choice (37, 38). Children select foods that are energy dense, familiar, or sweet (39). Although the nutritional composition of foods can be evaluated independently, their nutritional impact depends on how they fit into a dietary pattern (40). These are sensory questions, driven by the properties of the food we choose and the way we eat them. Sensory science supports this, showing that sensory properties shape acceptance and guide food choice and intake behavior, making taste a decisive driver of what people select and how much they consume. For manufacturers aiming to enable responsible consumption, this means that portion-guided products still need to offer an enjoyable experience. If taste is compromised, consumers may reject the product entirely or compensate by seeking satisfaction elsewhere, undermining the intent of portion control (41).

As highlighted by sensory studies, taste pleasure is not simply an emotion but a functional cue guiding eating behavior (40). Taste and texture can act as non-nutritive signals that shape how foods are eaten, influencing bite size, eating rate, and meal termination. Texture plays a central and often underestimated role in moderating intake. Texture literally instructs the eater how to eat, determining chewing effort, oral processing time, and swallowing rate. Slower eating, often a result of firmer textures, reduces energy intake and enhances satiety signals (40, 42). Consistent with these observations, there is ongoing research on how slowing the speed of eating can help manage calorie intake (43–45).

Studies suggest that sensory and physical properties of foods, particularly texture-related attributes such as aeration, hardness, viscosity, homogeneity, physical form, and added water, can reliably modulate satiation and subsequent energy intake without compromising consumer acceptability (46). Texture, in particular, has emerged as a consistent influence on food intake. Making snacks harder, chunkier, more viscous, more voluminous, or more solid can influence eating behavior. Manipulating the texture of snacks maintains sensory appeal and may be a viable, consumer-friendly approach to responsible snacking (46).

The sensory response to palatable snacks follows a pre-set time course. While the first few bites are most pleasurable, that pleasure may diminish as eating continues. Sensory specific satiety, a term developed and popularized by Rolls (47), refers to the observation that the perceived pleasantness of a given food decreases as more of it is eaten (48). This reduction in sensory pleasure operates within a single meal or even snack (48). Satisfying snacks may trigger sensory-specific satiety faster and so lower amounts of such foods are consumed. Flavor intensity is the important concept here (49). Sensory specific satiety could be an innate human mechanism to turn to other foods, seek greater dietary variety and so diversify the nutrients in the diet (25, 47). A related concept that “the better something tastes, the less you eat” (50) has its roots in a mix of philosophy, psychology, gastronomy, and ancient thought. The philosopher Epicurus believed that pleasure comes from moderation and that overindulgence can only lead to dissatisfaction. Anticipated palatability may drive snack selection, but sensory specific satiety aligns pleasure in moderation and may limit the amount actually consumed.

## 4 Structured consumption moments

Eating is an intensely private behavior that is often performed in public. The dichotomy between satisfying personal needs and obeying social norms is a recurring theme in anthropology and sociology (51). The concept of structured consumption applies to both individual snacking behaviors and to shared contexts. Structured consumption moments refer to discrete eating occasions, characterized by a clear start and end, sitting down, and minimal distractions. The consumer is more attentive to the eating experience by focusing on sensory stimuli that is the taste and texture of the snack. Slowing the rate of eating is one way to trigger satiation and so reduce calories consumed at this or even at the next snack (52).

Evidence on the impact of structured consumption comes from studies showing that paying attention to or reducing distractions during the eating event can lower immediate intake without compromising sensory enjoyment (53, 54). Those principles seem to apply to meals as well as to snacks. Importantly, planning a snack was more conducive to making healthier choices than were unstructured, opportunistic eating occasions (55). The concept of structured consumption moments may be particularly relevant for palatable energy dense snacks.

Social norms may also play a role in promoting responsible snacking. Commensality, or the act of eating together, has long served to create or maintain social bonds. The recent World Happiness Report 2025 has provided evidence that the act of sharing meals was closely linked to enhanced wellbeing and stronger social ties (56). People who frequently shared meals with others consistently reported higher life satisfaction and lower levels of loneliness, regardless of cultural or demographic background. A recent paper suggests that among all chocolate-elicited emotions, the intensity of “happy” was the highest. In other words, published research shows that consumers tend to associate chocolate with happiness (57).

Much of the traditional research in this area has pointed to the social facilitation of eating, leading to higher food consumption at meals (58). However, most of the 42 studies cited by Ruddock et al. (59) were based on re-analyses of food diaries and self-reported meal size and very few involved energy-dense snacks (e.g. cookies, crisps, chocolate). Whereas some studies found an effect, others did not. In other cases, the effect was equivocal; children ate more cookies only when paired with siblings but not other companions. In summary, there is no strong evidence for social facilitation of snack consumption. The French sociologist Claude Fischler has an interesting perspective on commensality. He has proposed that eating together acts as a regulating force in food intake (50). In his view, structured food patterns in France have helped to manage calories more effectively than in the US where people are more likely to eat alone (50). He has now extended the concept of commensality to between-meal snacks, suggesting that sharing highly palatable foods, generally eaten in small amounts, can act through soft regulations to limit the amounts of snacks consumed (Fischler, personal communication). Previously excluded (50), snacks have now gained a place in the commensality framework. Further research is needed to better understand commensality in the context of snacking, which may help inform how these eating occasions can be structured.

Fischler (50) has also argued that the act of sharing food activates a number of social-psychological mechanisms. Studies (58) have shown that people tend to model each other's eating behavior, adjusting their intake in response to social cues about appropriate consumption. People tend to moderate their consumption to avoid appearing inappropriate or deviant from group norms (60). The social visibility of sharing desirable foods creates a form of soft regulation, acting through the internalized desire to maintain a positive social image and conform to perceived group standards. This soft regulation helps to establish structured snacking moments, characterized by temporal boundaries (when snacking begins and ends), sitting down, and behavioral parameters (how much, at what pace) that are sometimes absent in solitary consumption.

## 5 The role of the food industry

The implementation of dietary guidelines requires multi-sector efforts. While consumer guidance is an integral component of food-based dietary guidelines, food manufacturers should also play a role. Food manufacturers hold a unique position in shaping consumption patterns through product formulation, packaging, portioning, and communication strategies. The present goal is to provide actionable, evidence-based guidance for manufacturers seeking to promote responsible snacking.

First, studies in experimental psychology suggest that limiting portion size is an effective way to manage consumption of energy dense foods (13, 19). Marketing studies confirm that providing individually packaged units, downsized packages, or clear portion indicators creates natural stopping points, simplifies decision making and assists with responsible snacking (30, 61). Reduced snack servings are generally acceptable when they are aligned with expectations and do not compromise perceived value (62).

Second, consumer acceptance depends on the sensory properties of snacks. Even within the 200 kcal/serving guidelines, taste and texture of snacks can enhance satisfaction, promote slower eating, and increase satiety at fewer calories (25, 63). Food manufacturers are responsible for product formulation and for the selection of ingredients. Efforts at reformulation have traditionally addressed added sugar and saturated fat; nutrient density of snacks can be improved by the addition of desirable ingredients such as whole grains, fruit, dried fruit, nuts, dairy, legumes, seeds and vegetables (38). The challenge is to improve nutrient density, manage portion size, maintain price point and not compromise on perceived value and sensory appeal.

Food manufacturers can play a role in facilitating structured snacking moments through product design, communication, and contextual cues. Portion-controlled packaging and single-serve formats can help define a clear beginning and end to the eating occasion, reinforcing awareness of quantity. Product on-pack messaging can frame snacks as intentional breaks, without invoking prescriptive or moralizing language. Sensory-forward product development may further incentivize slower, more attentive consumption by leveraging product characteristics that increase oral processing time, such as harder textures, increased chewiness, or larger volumes, thereby enhancing sensory engagement during eating.

Finally, manufacturers can support structured moments through usage suggestions that link products to specific contexts, aligning with evidence that when and how people snack matter as much as what they consume (55). For manufacturers operating in this category, they can help normalize low-distraction, structured consumption moments that support durable eating behavior.

## 6 Conclusion

This perspective has identified strategies for implementing responsible snacking that can be adopted by consumers and also by manufacturers in product development and communication strategies. Interestingly, the three laboratory strategies derived partially align with some manufacturer-led initiatives on product formulation, portion control, and communication strategies. The strategies also align with some priorities of the DGA 2025–2030, notably on portion control. Given the growth of the snack category, it becomes increasingly important to reconcile consumer demand with public health priorities. Future strategies to promote responsible snacking should embrace innovative product formulation, individually packaged units, clear portion indicators and guidelines for optimal snack use. Future research should further investigate how consumer behavior, expectations, and perceived pleasure interact with these behavioral principles in real-world settings. We also recognize that behavioral interventions are merely a part of a bigger picture: food systems are also subject to influence by regulations, taxation, other economic factors and the environment. To create meaningful and sustainable changes, manufacturers should review the quality of products on offer and share the responsibility for responsible snacking. People snack for energy and nourishment but also for enjoyment.

## Data Availability

The original contributions presented in the study are included in the article/supplementary material, further inquiries can be directed to the corresponding author.
